# The work of Donald Ewen Cameron: from psychic driving to MK Ultra

**DOI:** 10.1177/0957154X231163763

**Published:** 2023-03-25

**Authors:** Jordan Torbay

**Affiliations:** McGill University, Canada

**Keywords:** Brainwashing, Cameron, depatterning, MK Ultra, Montreal

## Abstract

Donald Ewen Cameron is known as the Canadian psychiatrist behind the Montreal Experiments, a series of brainwashing experiments. As part of a larger Central Intelligence Agency (CIA) project known as MK Ultra, the CIA regarded these experiments as a potential military weapon during the Cold War. However, a closer look into Cameron’s research and project MK Ultra shows that these experiments began long before Cameron was contacted by the CIA. Additionally, Cameron received funding for his experiments indirectly, so he was probably never aware the money was from the CIA. In this paper, I analyse the published work of Dr Cameron from the beginning of his career to his role in MK Ultra, and evaluate his own possible reasoning behind these experiments.

## Introduction

Donald Ewen Cameron is known for his brainwashing experiments at the Allan Memorial Institute in Montreal, which took place in the late 1950s and early 1960s. Also known as Subproject 68 of the US Central Intelligence Agency (CIA) project MK Ultra, these experiments played a significant role in the CIA’s quest to harness mind control as a weapon during the Cold War. The most well-known narrative around Cameron is that of an accomplice to military torture – a man who ‘sold his soul’ to the CIA and knowingly destroyed the lives of healthy patients. However, this may not be entirely accurate. According to Cameron’s published research, his theories of psychic driving began before he was even contacted (indirectly) by the CIA and originated as a possible treatment for mental disorders. Throughout Cameron’s career, much of his work was focused on searching for a cure for schizophrenia, and his ‘depatterning’ treatments that devastated many lives began with the same goal. Moreover, the CIA officers communicating with Cameron claimed to be from the Society for the Investigation of Human Ecology, so it is entirely likely that Cameron was never aware his research was intended to be used for military purposes. In addition, Cameron was one of the psychiatrists present at the Nuremberg trials to evaluate the mental capability of the accused. These trials served as a lesson to the world on the importance of ethical research practices and, like most people, Cameron concluded that the experiments were horrific. Thus, we are left with the question: how did Cameron become the mind behind such heinous experiments? In this paper, I shall give an overview of: Cameron’s published research; what made him the ideal candidate for MK Ultra; and finally, his own most plausible intentions as a psychiatrist and a researcher.

## Early work

Donald Ewen Cameron was born in Scotland, studied at the University of Glasgow, and lived in many different locations, including Maryland (USA), Zurich (Switzerland), Manitoba (Canada) and Massachusetts (USA), in that order. In 1935, Cameron published the book *Objective and Experimental Psychiatry*, in which he emphasized the importance of rigorous knowledge of biology when studying psychiatry, and highlighted the effects of the environment on an individual organism, citing both British and European schools of psychiatry. He worked as a researcher in Albany, New York, for many years, until the renowned neurosurgeon Dr Wilder Penfield invited him to work at McGill University in Montreal, Canada. In 1943, Cameron set up his research laboratory known as the Allan Memorial Institute in ‘Ravenscrag’, a mansion that formerly belonged to Sir Hugh Allan (Academic, 2022).

Dr Cameron always believed in a strict scientific method (Academic, 2022). In his published work, he always stated his research question, clearly outlined the materials and methods, and explained the results. His earlier work in the 1930s covered various topics in psychiatry, including epilepsy, depression, anxiety, emotion, and psychotropic drugs, but he took a particular interest in schizophrenia. Cameron examined many potential treatments for schizophrenia, as well as physiological differences between people with and without schizophrenia. In 1934, he published the paper ‘Heat production and heat control in the schizophrenic reaction’. Patients were placed in extreme heat: a room at 36–42º C for one hour, and their change in body temperature was measured. Response to cold was also measured: patients were placed in a tub of cold water at 28º C for 12 minutes, with their temperature taken every three minutes. Participants’ baseline body temperatures were measured, as well as body temperatures before and after ingesting food. Results showed that the average body temperature of schizophrenic patients was lower than that of controls, and it also fluctuated more. Schizophrenic patients responded more dramatically to extreme cold but were no more affected by extreme heat than were controls (Cameron, 1934).

This 1934 study is a prime example of how ethics can be neglected when researchers are set on finding answers. Being exposed to 40º C heat for an hour could put a person at risk of heat exhaustion, and it would certainly not be pleasant to endure. Another paper published by Cameron (1931) examined the effects of dehydration on epileptic patients. They were put on a low-water diet where they were allowed as little as 600 ml of water per day, and even given diuretics to decrease water retention. Results showed no significant differences in number of seizures between the low-water group and the control group. However, it was observed that patients would become so desperate for water that they would steal food and drink, even attempting to drink out of flower vases and consume snow from windowsills. Patients experienced severe weight loss and acidosis from increased blood urea nitrogen. One patient, known only as ‘Patient 11’, died during the experiment.

## Nuremberg Trials

When we think of unethical experiments, what immediately comes to mind is the Nuremberg Trials after World War II. One of the psychiatrists present at the Nuremberg Trials was Dr Ewen Cameron himself. He was sent, along with other British, French, American and Soviet psychiatrists, to evaluate the mental state of Rudolf Hess (Academic, 2022), Deputy Führer of the Nazi party and a fanatically loyal underling of Adolf Hitler. Hess had been a part of the German military since the age of 20 during World War I and had been in awe of Hitler since hearing him speak publicly. Hess first served as Hitler’s secretary and was then appointed Chairman of the Central Political Commission of the Nazi Party and SS General in 1932, before eventually being named Deputy Führer, a mainly ceremonial position, in 1933. Hess has many times been described as blindly obedient and loyal to Hitler, comparable to religious fanaticism. When captured by the British, he was declared mentally unstable, and even Hitler deemed him insane (The History Place, 1996). At the Nuremberg Trials, Hess was evaluated by multiple psychiatrists to ensure that he was mentally competent to stand trial.

According to Dr H Lehmann, patients’ insanity levels should be classified I–IV, with I being normal and IV being acutely unstable. Lehmann classified Hess as IV-Psychotic, and described him as having ‘[i]mpractical ambition, impulsiveness, reasoning based on symbolism rather than logic, confabulation with a tendency toward the bizarre, as well as a rigid egocentric attitude’, traits that point to ‘schizophrenic personality disintegration’ (Lehmann, n.d.). However, when Cameron examined Hess, he observed that his symptoms of memory loss were inconsistent with those of a normal amnesiac. He concluded that ‘a part of the memory loss is simulated and it is probable that the hysterical or unconscious part is rather superficial’, saying that this pretence of amnesia was ‘originally developed consciously as a protective measure’, but had ‘become unconscious in part’. Furthermore, Cameron described how Hess later admitted on trial ‘that he had simulated his loss of memory for tactical reasons while in England but he had intended to reserve his declaration for a later period in the trial’ (Cameron, Lewis and [Schroeder], n.d.). While Hess showed other signs of ‘great instability’ (Cameron et al., n.d.), he was ultimately declared fit to stand trial, and subsequently sentenced to life in prison. At the age of 92, Hess committed suicide in prison (The History Place, 1996).

History repeats itself: there are many distinct similarities between the World War II experiments prosecuted at the Nuremberg Trials, and Cameron’s experiments. They were both conducted during periods of political unrest; many of Cameron’s experiments took place in the 1950s when facing the threat of a nuclear war. Among the World War II experiments were extreme cold experiments at Dachau, where victims were placed in vats of cold water or outside in extremely cold conditions, while experimenters recorded their physiological reactions (Tyson, 2000). Cameron also used temperature as an independent variable in his 1934 study with schizophrenic patients, using extreme heat and cold on participants (Cameron, 1934). Also at Dachau concentration camp, experimenters attempted to find a way to make sea water drinkable, forcing victims to drink sea water while depriving them of food and fresh water. Victims became severely dehydrated to the point where they licked floors that had just been mopped in attempts to get drops of fresh water (Tyson, 2000). Similar desperate behaviours were observed in Cameron’s (1931) study on the effects of dehydration on epilepsy, mentioned above. However, the World War II experiments measured physical responses to various illnesses and injuries, whereas Cameron’s infamous experiments in Montreal were primarily psychological. As reported in Academic (2022), Cameron – like the vast majority of people – found the World War II experiments horrific; he published a paper titled ‘Nuremburg and its significance’, where he described the atrocities of these experiments and what it meant for the German people. He also gave a lecture called ‘Dangerous men and women’, where he concluded that the German race was inherently predisposed to inhumane acts. Cameron differentiated populations into the ‘weak’ and the ‘strong’, expressing his concern at the ‘weak’ continuing to reproduce (Academic, 2022). The Nuremberg Trials brought to light the dangers of experimenters disregarding the well-being of subjects and, after that, scientists across the world began to prioritise physical health and safety of their patients. However, the same cannot be said for patients’ mental well-being.

## MK Ultra

During the Cold War Era of the 1950s, the possibility of a nuclear war with the Soviet Union was such an existential threat that the CIA was determined to make use of the most powerful weapon that they could: the human mind. Mind control was not new to the CIA – in the early 1950s, Project Bluebird was already testing the waters. When Sidney Gottlieb was hired to the CIA in 1951, his supervisor, Allen Dulles instructed him to advance the field. Project Bluebird was expanded and soon became known as Project Artichoke, led by Gottlieb. In the early 1950s, the CIA believed that the Russians and the Chinese already had a handle on mind control techniques, citing brainwashing to explain their immense power over their citizens. Determined to keep up in the arms race, American researchers led by Gottlieb began experimenting with interrogation techniques and use of psychotropic drugs, much of which would now be classified as torture. After extensive research into mind control techniques, Gottlieb set out to establish a bigger, more ambitious project to discover the secrets of ‘brain warfare’, once and for all. In 1953, Gottlieb’s proposal was approved by Dulles, which was from then on known as MK Ultra (Kinzer, 2019: 99).

Throughout the search for the secret behind mind control, many people from different backgrounds were recruited, including Dr James Hamilton, Dr Robert Hyde (p. 100) and Dr Louis Jolyon West (p. 88) who were all American psychiatrists. Hamilton worked at Stanford University, and he became the researcher behind Subprojects 2, 124 and 140. Subproject 2 examined how synergistic activity of drugs can eradicate consciousness completely, as well as ways to drug a person without their knowledge. Subproject 124 tested whether inhaling carbon monoxide gas would induce a trance, and Subproject 140 tested the psychological effects of thyroid hormones (p. 99). Hyde coordinated Subprojects 8, 10, 63 and 66, which studied the effects of psychotropic drugs, in particular LSD-25 and alcohol (p. 100). West was paid $20,800 by the CIA to test the effects of dissociative drugs (p. 88). As drugs were a key area of research, pharmacies and pharmacologists were also key allies. The American pharmaceutical company Eli Lilly was behind Subprojects 6 and 18, where the CIA paid $400,000 for bulk purchases of LSD, making Subproject 18 the most expensive subcontract (pp. 86, 130). Dr Harris Isbell was an American pharmacologist who studied the effects of LSD, mescaline, psychotomimetic drugs (that cause delusions) and even worked on the pre-clinical development of new drugs (p. 95). Another recruit was Dr Carl Pfeiffer, an American physician and biochemist who studied depressant and hallucinogenic drugs (p. 97).

Not everyone recruited was from traditional backgrounds. One of the most well-known figures was George Hunter White, who held the title of Federal Narcotics Agent and CIA Consultant under the pseudonym Morgan Hall. In temperament, White was vastly different from many of the CIA’s allies; he had a sadistic streak and was well-versed in the illegal underground. He gained visibility for exposing a Chinese-American opium ring, having been initiated into the inner circles and facing ‘death by fire’ should he betray them. At first impression, White was rather rough, being well acquainted with criminals, pimps and prostitutes, and having a taste for sadomasochism. Despite their vast differences, White and Gottlieb soon became close comrades. White became known as the person behind Operation Midnight Climax, also known as Subproject 35. As per Gottlieb’s instructions, White enlisted prostitutes to lure clients to their ‘safe houses’, where they were drugged without their knowledge so that CIA psychologists could study the effects of sex and drugs on the human psyche (Kinzer, 2019: 149–51).

Another ally of the CIA, John Mulholland, was not a scientist at all but a magician. He was a student of Harry Houdini, an author of several books, a renowned illusionist and a skilled performer. One of his areas of mastery was the ‘psychology of deception’, making him a valuable ally to the CIA. Gottlieb enlisted him to write a manual on how to use magicians’ stage techniques for distraction and deception, intended for CIA officers to use in practice. This original project known as Subproject 4 was expanded into Subproject 19, until the final version was published: ‘The Official CIA Manual of Trickery and Deception’ (Kinzer, 2019: 88, 92).

At the 1954 convention of the American Psychological Association, Gottlieb’s officers were drawn to a Canadian study by Dr Donald Olding Hebb at McGill University, testing the ‘effects of radical isolation upon intellectual function’. Hebb was dubbed the ‘father of neuropsychology’ for his innovative research that combined neuroscience and psychology. The well-known Hebbian theory of neural plasticity stated that connections between neurons become stronger and more sensitive when they are activated more frequently. In other words: ‘Cells that fire together, wire together’ (Ferguson, 2022). Like Cameron, Hebb conducted psychological experiments. His participants were McGill students who volunteered in exchange for $20 compensation per day of the experiment. In these sensory deprivation experiments, the participants lay on hospital beds with translucent goggles, arms cuffed, and speakers that emitted buzzing noises in their ears. After three days of this treatment, participants experienced surreal hallucinations (Hebb and Heron, 1955).

After further investigation into Dr Hebb’s research, CIA officers became even more interested in a colleague of Hebb: Donald Ewen Cameron. He adapted Hebb’s experiments using a greater degree of force, using drugs and hypnosis to induce a ‘clinical coma’, and he compared the subjects’ psychological breakdown to criminals breaking down under interrogation (Kinzer, 2019: ch. 8). The longest any of Hebb’s patients lasted was six days of sensory deprivation (Hebb and Heron, 1955), whereas some of Cameron’s sensory deprivation studies lasted up to 35 days (Kinzer, 2019: ch. 8). Within the next few years, Cameron would become Gottlieb’s most valuable ally in the quest for mind control research.

## Psychic driving and depatterning

In 1953, Cameron developed the theory of ‘psychic driving’ (Simkin, 2020), where the minds of patients were manipulated using verbal cues played repeatedly. The objective was to bring repressed thoughts to the forefront of the patient’s mind such that they could identify them. These cues could either be recordings of the patient’s own statements (‘autopsychic driving’) or spoken by an experimenter (‘heteropsychic driving’). In a study by Cameron (1956), a high-fidelity magnetic tape recorder was used to record a 40-year-old female patient’s own verbal cues. She was then forced to listen to her own emotionally charged statement: ‘If you don’t keep quiet, I’m going to leave you behind’, a statement originally said to her by her mother, that stayed with her all her life. Over the course of 45 repetitions, the patient expressed her distress, begged the experimenter to stop playing it, turned red and began to hyperventilate, started shaking and continued to shake after the recording was stopped. In this study, heteropsychic driving was also used on patients for 10–12 hours, sometimes during sleep. Sodium amobarbital was used to put patients in a ‘clinical coma’, in order to extend the periods of psychic driving for up to 20 hours a day, over a period of 10–15 days. In this paper, Cameron cited Hebb’s ‘psychological isolation’ techniques to decrease defence mechanisms, where a patient was placed in a dark, quiet room with goggles covering the eyes, and prevented from touching his or her own body as a way of ‘interfering with his self image’ and decreasing his ‘expressive outflow’. Cameron also experimented with stimulant drugs such as Desoxyn and mentioned the possibility of using hypnosis.

Throughout these experiments, Cameron carefully observed patients’ responses, and identified six major classes of responses. A 50-year-old female patient, listening to the recorded statement ‘That’s what I can’t understand – that someone could strike at a little child’, showed an ‘immediately constructive response’, i.e. severe distress after 10 repetitions. A 28-year-old woman listening to a statement based on her childhood difficulties with her father showed a ‘partial block’; according to Cameron, the ‘intensity of feelings’ from the experiment led to a reduced ability to communicate with her therapist. The ‘rejection and later acceptance’ response was supposedly common among patients put under prolonged periods of sleep, where patients ‘reject’ the statement for up to 10 days before internalizing it. A young woman demonstrated the ‘rejection and escape response’ after listening to a statement on her supposed sexual desire for her father: she became angry and hostile towards the therapist, then quit therapy until she was later admitted to in-patient treatment, ‘deeply disturbed’. The ‘continued action’ response was seen in an anxiety patient, who, after listening to a ‘reassuring statement’ over a period of eight days, stated that she found reassurance in this statement when she felt afraid. Finally, the ‘development of defences’ as a response was seen after long periods of heteropsychic driving, including a woman who underwent a minimum of 25 hours of psychic driving, part of it under LSD-25. She later stated that she could not remember certain themes of the psychic driving sessions, such as giving up drinking, and instead had an urge to drink, which Cameron described as an ‘inversion of one of the heteropsychic driving themes’ (Cameron, 1956).

In another paper, Cameron (1957) described psychic driving as having the ability ‘to activate and bring progressively into his awareness more recollections and responses generally from this area [of the repeated statement]’. While he claimed the end goal to be ‘the accelerating of therapeutic reorganization’, most of the patients instead experienced a form of mental ‘depatterning’, leaving them dysfunctional. This experiment took place in 1954–5 and involved 10–30 minutes of autopsychic driving: a patient’s own recorded statement of roughly 20 seconds (described as a ‘dynamic implant’) was recorded on a magnetic tape, stored on a 14-inch record, and played to the patient via a high-fidelity phonograph. Patients showed a great deal of distress; one patient came into the therapy session in a pleasant mood, with nothing she wished to talk about, but throughout the 10 minutes of psychic driving, became ‘restless and anxious’, begging the experimenter to stop. When asked how she felt afterwards, she answered: ‘It made me nervous all over again. Everything hurt me all over as it did before. My voice sounds like I am going to die.’ This paper claimed that out of all psychic driving experiments, ‘In only one has there been seen a possible persisting trauma resulting from the implant’.

Experimenters began using headphones on patients to play the statement for ‘greater impact’, and patients compared it to hearing a voice in their own head. Experimenters also created a filtered record of the original recording, with particular emphasis on treble notes, bass notes, low-volume sounds, high-volume sounds or an ‘echo-back into the communication’. When listening to the original recording, one patient showed ‘no response’, and afterwards stated she ‘had no feeling at all as [she] listened; [she] was thinking of something else’. In contrast, after listening to a ‘filtered’ record, she said: ‘I feel like screaming and putting on a tantrum. The voice seems to scream at me all the time. . . . I feel trapped. I feel I can’t talk to anyone.’ Cameron concluded that psychic driving was supposedly most effective during the last 10 minutes of the therapy session so the effects were not interrupted; he also considered the possibility of spending five minutes after the psychic driving asking the patient about his or her experience, which according to Cameron ‘probably tends to stabilize the implant’. He observed that the dynamic implant caused ‘mobilization of action tendencies’; the patient would become aware of the underlying issues expressed by the repeated statement which ‘facilitates problem identification by the patient and the therapist’. Cameron also noted that the effects of the dynamic implant were dependent on five factors: the number of repetitions, the ‘intensity of response’, the patient’s defence mechanisms, the patient’s own tolerance to stress, and ‘capacity for desensitization’, i.e. how effectively patients could desensitize themselves to the implant (Cameron, 1957).

In January 1957 (Simkin, 2020), Gottlieb launched Subproject 68 of MK Ultra, in which the CIA began subsidizing Cameron’s experiments, giving him $69,000 over the next few years. As a front, the CIA sent him funding via the ‘Society for the Investigation for Human Ecology’, classifying these experiments as civilian research in the eyes of Cameron and his colleagues (Kinzer, 2019: 139). As well as psychic driving, a brainwashing technique known as ‘depatterning’ reduced patients’ minds to an infantile state so that they could then be rebuilt according to the wishes of the experimenter. A paper by Cameron and Pande (1958) reported this treatment on 26 paranoid schizophrenic patients, 16 with symptoms for two years or more and 10 with symptoms for less than two years. Cameron cited a 1935 German paper by M. Sakel claiming that ‘a prolonged and severe so-called reversible coma’ may be an effective treatment for cases that did not respond to other forms of treatment. Chlorpromazine and various barbiturates were used to induce a ‘prolonged sleeping state’ for 20–22 hours per day, over a period of 10 days. After that, electroshock treatments began with the goal of ‘complete depatterning’, which developed in three stages. In the first stage, the patient had noticeable memory loss, but was still aware that he was in a hospital and could recognize doctors, nurses and family members. In the second stage, the patient had ‘lost his spatial and temporal image’, but was conscious of these defects, asking questions such as ‘Where am I?’ and ‘How did I get here?’ while also showing difficulty with basic motor skills, and incontinence. In the third stage, ‘his original delusional ideas’ and ‘all other evidences of his schizophrenic behavior’ were ‘completely absent’, but at this point, the patient’s mind had become an entirely blank slate (Cameron and Pande, 1958). Cameron’s logic is not wrong: the delusions and hallucinations specific to schizophrenia have been eliminated, but so have virtually all other forms of cognition. Additionally, chlorpromazine was at that time being discovered as an effective treatment for manic or hypomanic states, according to a 1954 paper by Lehmann and Hanrahan (see also Swazey, 1974: 153). It was likely the chlorpromazine rather than the electroshock treatments that ‘cured’ Cameron’s patients of their positive symptoms of psychosis, a fact Cameron may have known since his paper was published three years after Lehmann’s.

Afterwards, the ‘phase of rehabilitation’ would begin, in which the patient was closely monitored by a caregiver and, if schizophrenic symptoms returned, ‘electroshock is again intensified’. Cameron and Pande (1958) note that it is necessary to ‘distinguish actual schizophrenic relapses from forms of behavioural disturbance’, presumably as a result of the depatterning procedure, which occurred in approximately 25 per cent of patients. These symptoms were treated with chlorpromazine or reserpine. After one month of ‘rehabilitation’, patients were discharged and given frequent follow-up electroshock treatments. Patients were given psychotherapy during the rehabilitation stage and the two-year follow-up, with the goal of helping the patient to become ‘stabilized’ and return to normal functioning. This paper claimed that this was an effective treatment; out of 16 patients with schizophrenic symptoms lasting two or more years, all 16 of them were discharged and 11 had ‘good’ results at follow-up appointments, though ‘some residual evidence of schizophrenia’ was noticed. However, two patients developed ‘paranoid reactions’ to follow-up care, and three were re-admitted to the hospital, of which two were later discharged and one was still being treated at the time of publication. Cameron concluded that total depatterning was the most beneficial treatment for severe cases, claiming that ‘recovery consists not in repair of damaged aspects of the individual’s personality but in a rearrangement’.

These experiments went on for many years. Cameron, Levy, Rubenstein and Malmo (1959) studied ‘psychoneurotic’ patients, including those with chronic anxiety, obsessive compulsive disorder, schizophrenia, and seemingly minor issues such as ‘passive aggressive personality’, ‘aggressive type’ and ‘inadequate personality’. The procedure consisted of 30–60 days of induced sleep during which recorded statements, mostly positive and encouraging, were played, followed by prolonged sleep and ‘intensive’ electroconvulsive therapy (ECT) in cases where ‘pathological behaviour was strongly structured’, followed by bromide administration to induce delirium before playing the recording. Results showed consistent ‘verbal acceptance’ of the recorded messages, as well as ‘acting out the content’ of the messages. In a further study by Cameron, Levy and Rubenstein (1960), phencyclidine (PCP), a drug which ‘blocks extraneous sensory input’, was given along with chlorpromazine and barbiturates to put the patient in ‘a passive receptive state with a heightened awareness of the verbal signals’. Both positive and negative verbal signals were delivered via speakers in patients’ pillows. This paper included an acknowledgement of a grant from the Society for the Investigation of Human Ecology, Inc., the cover that the CIA was using.

In another study, examining induced amnesia as a treatment for schizophrenia, Cameron (1960) criticized the use of the term ‘regressive shock therapy’ for his depatterning procedure, saying it evoked an ‘actual functional return to infancy’ as opposed to ‘merely a partial similarity in behavior’. This study, which included 53 schizophrenic patients, followed the same procedure of induced sleep and intensive ECT, but with the goal of inducing amnesia to the point where ‘the patient loses all recollection of the fact that he formerly possessed a space-time image which served to explain the events of the day to him’. For a period of time, the patient ‘lives in the immediate present’ with a conceptual span ‘limited to a few minutes and to entirely concrete events’, but is gradually allowed to ‘re-emerge’ from this amnesia. Cameron ultimately concludes that the patient ends up with ‘a considerably greater amnesia for his schizophrenic behaviour than for his concurrent normal behaviour in the period prior to treatment’. In the next paper, on sensory deprivation (Cameron, Levy, Ban and Rubenstein, 1961b), Cameron cites his colleague Donald Hebb’s sensory deprivation experiments in the 1950s. He then introduced his own research at the Allan Memorial Institute where ‘psychoneurotic and schizophrenic’ participants were kept in a soundproof room with rubber eardrums emitting ‘white sound’, goggles obscuring their vision, cardboard cylinders over their arms, and no social interaction except when interviewed by researchers or ‘fed and toiletted’ by nurses. Although participants were told they could leave the study at any point, experiments lasted up to 16 days, and patients experienced hallucinations and depersonalization as a result of ‘repression and aggression turned against the self’. Patients were also given drugs such as Cl-400 and PCP, the latter of which causes apathy, anxiety, and disturbance of body image.

By 1961, Cameron had made six key conclusions which he summarized in his paper for the *Canadian Psychiatric Association Journal* (Cameron et al., 1961a): (1) the key component of the psychic driving theory: verbal signals lead to changes in behaviour; (2) these statements can be set up by the experimenter; (3) a patient’s ‘personality set’ can be ‘temporarily broken up’ by means such as induced sleep and electroshock therapy; (4) personality changes are more effectively induced when targeting a ‘dormant behavioral pattern’; (5) repeated verbal signals can cause physiological changes as well as psychological ones, such as the flexor–extensor dominance of forearm muscles; and (6) these methods are more effective when follow-up psychic driving sessions are provided for weeks or months after the initial procedure. With these premises in mind, this study examined 10 ‘psychoneurotic’ patients undergoing the psychic driving procedure. In this study, they were given 15–150 mg of curare in beeswax to decrease activity, along with sensory deprivation. Cameron concluded that this treatment led to positive changes for the patients’ mental health and functioning.

A paper in the following year (Cameron, Lohrenz and Handcock, 1962) described a study of schizophrenic patients who were given three barbiturates – Veronal, Seconal and Nembutal – as well as chlorpromazine to keep them asleep except for being wakened three times daily for meals and to use the toilet. This study also introduced a new monoamine oxidase inhibitor, RO4-1038, to alleviate ‘organic anxiety’ from the procedure and to prevent the patient from becoming ‘seriously disturbed’. In accordance with the conclusions of the 1961 study, patients were given follow-up ECT appointments for 22–68 months. In this paper Cameron criticizes the name ‘regressive shock therapy’, citing a 1948 paper by Kennedy and Ancell for this ‘misleading designation’. The next paper (Cameron, Levy, Ban and Rubenstein, 1964) commended the possibility of ‘automation of psychotherapy’, saying it offered an alternative to the ‘time consuming’ process of psychotherapy. This paper claimed that psychic driving had many positive consequences for the patient, including uncovering underlying causes of psychological problems, identifying problems, desensitization to emotional distress, patient–therapist interaction, problem-solving, and reorganization of thoughts and beliefs.

## Aftermath and discussion

Dr Cameron is widely known as a ‘CIA doctor’, but it is debatable to what extent his work was influenced by the CIA. Since his early research in the 1930s, Cameron had taken an interest in schizophrenia and its potential treatments; his 1934 paper on his study using extreme heat and cold aimed to better understand the differences between schizophrenic patients’ and control subjects’ physiological responses to external stimuli. When the CIA began funding Cameron’s experiments at McGill, he was supposedly unaware that the money came from the CIA. Cameron signed a contract in 1957 outlining the research he agreed to oversee:

(1) The breaking down of ongoing patterns of the patient’s behavior by means of particularly intense electroshocks (depatterning). (2) The intensive repetition (16 hours a day for 6–7 days) of the prearranged verbal signal. (3) During the period of intensive repetition the patient is kept in partial sensory isolation. (4) Repression of the driving period is carried out by putting the patient, after the conclusion of the period, into continuous sleep for 7–10 days. (Kinzer, 2019: ch. 8)

This is essentially the same as the research he was already doing. According to Cameron’s published work, there was no noticeable change in direction of the experiments before and after the contract was signed.

To illustrate this, let us compare two of Cameron’s papers: one published before and one after the grant. The first paper, titled ‘Psychic driving’, was published in 1956 and set out to measure the effects of the repeated playback of verbal cues. Some patients were given amobarbital, some were given LSD-25 and, with some patients, psychic driving took place during ‘prolonged sleep’ and sensory deprivation (Cameron, 1956). The second paper, titled ‘Effects of repetition of verbal signals upon the behaviour of chronic psychoneurotic patients’, was published in 1960 after Cameron had signed the contract with the CIA via the Society for the Investigation of Human Ecology, the cover the CIA was using to fund Cameron’s experiments. This paper acknowledged a grant from the Society for the Investigation of Human Ecology (Cameron, Levy and Rubenstein, 1960). Although patients were given a slightly different combination of drugs from those used in the 1956 study, both studies essentially had the same research question: how can repeated verbal statements, along with drug administration, affect patients’ behaviour? Both studies involved drugs but were mainly focused on the effects of the recorded statement. Both studies used statements played via speakers in patients’ pillows, and both studies had similar lengths of psychic driving sessions: the 1956 study for 10–20-hour periods, and the 1960 study for 16-hour periods. Finally, both studies used patients with a variety of mental disorders and concerns, not just schizophrenia, and both studies ultimately caused far more harm than they claimed to fix.

Despite the claims that Cameron’s experiments were a legitimate medical treatment, these experiments had devastating impacts on patients and their families. In a Canadian Broadcasting Corporation (CBC) podcast, Allan Tanny described how his father Charles Tanny, a hardworking man with trigeminal neuralgia, was admitted to the Allan Memorial Institute and was never the same after he returned (Shephard, 2020–21). In the following decades, numerous lawsuits were filed on behalf of Cameron’s former patients, including Tanny. In 2019, a class action lawsuit was filed in the Superior Court, District of Montreal: J. Tanny vs. Royal Victoria Hospital et al. (Consumer Law Group, 2019). Charles Tanny’s daughter Julie Tanny filed the lawsuit on behalf of her father and other victims of the Montreal experiments, seeking compensation for victims and their families. The legal proceeding described how Tanny was given over 50 days of insulin-induced ‘sleep therapy’, in which he was given multiple drugs: barbiturates (Seconal, Nembutal, Veronal, Amobarbital), antipsychotics (Sparine, Reserpine, Chlorpromazine) and glutethimide, a hypnotic sedative. He was also given frequent ECT treatments, many of which were Page-Russell, an intensive form of ECT where shocks continued during convulsions. After his treatments, Tanny experienced near total memory loss and disorientation, as well as incontinence, symptoms which never completely disappeared. Returning home from the hospital, Tanny had changed from being a loving and engaged father to emotionally distant and volatile, even physically abusive towards Julie Tanny, a young child at the time. This class action lawsuit entitled all of Cameron’s former patients to compensation for their injuries, and all family members and dependants to compensation for loss of support and emotional trauma as a result of their relationship with the patients.

Cameron’s experiments had devastating consequences for patients and their families. What began as hopeful treatment for severe mental illness strayed from legitimate medical treatment and became a form of medical torture likened to the Nuremberg Trials. Hence, how could a psychiatrist present at the Nuremberg trials, who condemned the entire German people for allowing such experiments to happen, become involved in such experiments? In reality, Cameron’s experiments began with the aim of treating schizophrenia, with no political or military purposes, and even after the CIA began funding the experiments, Cameron was kept mostly in the dark about where the money came from. There is no evidence showing Cameron wished to be part of any CIA project; it is more plausible that he was driven by sheer intellectual curiosity like most scientists. While modern medicine shows that these experiments have little scientific value, much of Cameron’s logic was sound: his experiments showed a clear research question, methods, results and interpretation. This does not excuse the lack of regard for patients’ welfare; ethics must always be at the forefront of research, something Cameron would have been aware of, having been present at the Nuremberg trials. However, many scientists, including colleagues of Cameron, have done ethically controversial studies. Instead of the false dichotomy of the ‘good’ scientists versus the ‘bad’ scientists, we must be aware that we are all capable of such atrocities if we allow our desire for answers to come before our first duty: to do no harm.
